# Assessment of leakage management in small water supplies using performance indicators

**DOI:** 10.1007/s11356-021-13575-5

**Published:** 2021-03-29

**Authors:** Iwona Klosok-Bazan, Joanna Boguniewicz-Zablocka, Aneta Suda, Ewelina Łukasiewicz, Dorota Anders

**Affiliations:** 1 Department of Thermal Engineering and Industrial Facilities, Faculty of Mechanical Engineering, Opole University of Technology, Opole, Poland; 2 Department of Renewable Energy, Institute of Technology and Life Sciences in Falenty, Poznań, Poland

**Keywords:** Performance indicators, Water losses, Drinking Water Directive, Leakage management

## Abstract

The revision of the Drinking Water Directive (DWD), which the process of consultation by the European Parliament is coming to the end, includes a commitment for all Member States to assess the level of water leakage. The overarching aim of this action is to reduce water loss through leaks. In regard to this, use of performance indicators as suitable to enable assessment of water utility performance with regard to leakage reduction, including impacts on environment and cost-efficiency, is recommended. The paper provides results from water losses evaluation with the set of performance indicators (PI), considering dependence on the availability of resources and specific operating conditions. An analysis of different PI that are necessary to evaluate in most of leakage reduction methods was conducted considering step-by-step approach. Furthermore, the most relevant data for leakage analysis and management on a network level was determined. Presented PI can be used to improve leakage management of small water supply system. Similarly as in the Deming cycle, the described tool provides for planning activities, executing activities in accordance with the plan, checking the degree of execution of objectives included in the plan, and on the basis of conducted analyses and lessons learned, the so-called improvement. The results of the analysis lead to the conclusion that through a comprehensive, systematically improved strategy, the high effectiveness of the system could be reach. The reliable monitoring does not have to be based on advanced technological methods, which are often unavailable for small water supply system, due to high investment and operating costs. The results indicate that integrated IT systems, as SCADA, might not be directly beneficial to water loss management in small networks with moderate leakage levels.

## Introduction

With increasing population, the need for research ideas on the field of reducing wastage of water can save a big amount of water, money, time, and energy. The recently agreed revision of Drinking Water Directive (DWD) (Provisional agreement 2020) is taken this into the account. Currently, drinking water is controlled “end-of-pipe.” The newly agreed rules implement the so-called risk-based-approach, allowing for further prevention and mitigation measures to protect drinking water sources. New Drinking Water Directive includes an obligation to assess water leakage levels and to reduce leakages in case they are above a certain threshold. High levels of water losses in distribution systems (both real and apparent losses) continue to burden water companies and customers around the world: water losses vary from very low to unsustainably high (AL-Washali et al. 2020). Pressure management and fixing leaks to reduce real (physical) losses are part of a straightforward way of addressing water quality issues, as well as addressing water conservation. Despite much progress being towards addressing water losses in distribution systems, losses continue to exceed economic levels (Cavazzini et al. 2020). The basis for making decisions about commencing efforts to reduce losses or their elimination is the balance of produced and sold water. Based on annual water balances, a group of indicators need to be developed that describes water losses and helps to monitor and provide the necessary information for proper distribution network management (Alegre et al. 2016). These activities are considered to be cost effective if the monetary value of the water losses exceeds the cost of their disposal. In order to determine the economic level of leakages, it is necessary to conduct an economic balance sheet, which consists of the costs of active control and removal of leakages, water intake, treatment, and distribution, as well as the development of additional water resources. It is recommended that the acceptable level of leakage be set in a broader context than the economics of the water utility. Water loss should include the resource and environmental costs of lost water, as well as other costs resulting from leakages (Moslehi et al. 2020). They may result from subsidence of buildings or road collapse or traffic jams caused by the removal of water supply failures. The economic level of leakage is also influenced by the proportion between variable and fixed costs of producing drinking water. A significant share of fixed costs is characteristic of oversized water supply systems (WSS) and affects the establishment of a high level of losses, which is not economically viable. Limiting water losses not only reduces costs but also protects consumable water resources, which are only around 3% in the world (Iwanicka 2007). The main reason for initiating measures aimed at limiting water losses is economic factors. Currently, Polish law does not contain elements that would support start constraints. Their limit value is not established. Water and sewage companies are also not obliged to manage water losses. Often the pressure to limit them is exerted by city or municipal councils that want to keep water prices as low as possible. Counteracting water losses requires managing them, inter alia, by determining the amount of actual losses, setting acceptable levels of losses for the areas served and their maintenance. Start lowering effects up to a dozen percent are easily achieved. Their further reduction is much more difficult and more expensive. In order to achieve satisfactory water losses, enterprises should conduct continuous and systematic activities (Piechurski 2014). The most important activities to reduce water losses include installation of water meters and check valves and their control, network pressure control, modernization of water supply networks, and beforehand optimal design of water distribution network; implementation of water leakage elimination programs in water utility companies; and dissemination of water loss reduction concepts (Suribabu 2017). Guidance documents, published by the Commission, on how to reduce water supply losses emphasize that one of the important factor causing water loss is the material from which the water pipes were made and their age (EU 2015). In this case, actions aimed at limiting water losses mainly include inspection of pipes and fittings and their systematic repair or replacement (Bergel and Pawełek 2006). As a result, pipes with increased resistance to damage and changes in structure over time are used more often. They are mainly thermoplastics such as PE and PVC, multi-layer ducts coated with polyester resins, and ductile iron. In Poland, cast iron is currently the dominant material. Enterprises serving less than 20,000 inhabitants have the highest share of plastic pipes. Probably due to the fact that the implementation of investments is based on tenders in which the most important criterion for selecting offers are costs. This may contribute to the use of cheap pipes with inferior fault resistance parameters. This, in turn, may undoubtedly make it difficult to counteract the increase in water losses (Ociepa 2016).

The WSSs are categorized on the basis of their sizes to efficiently perform their organizational, financial, human resources, and operation and management (O&M) activities (Haider et al. 2014). In Poland, the biggest problems with WSS management have small WSS, the infrastructure of which was made in the 1950s, often with the effort of the inhabitants of the local community, without due engineering care. As a result, water utilities adopt various strategies for reducing water losses depending on the situation. Two types can be distinguished among them: passive and active. Passive water loss management is limited only to removing reported failures; then, the plant does not conduct a systematic policy and active leak detection. Such a strategy is undertaken mainly by enterprises where the cost of leak detection is high and the costs of water production are relatively low. Active management is based on continuous monitoring of network flows, which ensures continuity of supply and often a very large reduction in water losses. There are three basic issues in water network monitoring, such as active leakage control, speed of repairs, and pressure management in networks. The data obtained from the monitoring allow to observe and analyze water losses in a specific area, control the pressure in the network, and show the technical condition and basic parameters of the network. The operator can obtain a lot of information about actual water losses thanks to field monitoring, among others, through measurements and analysis of flows and abstraction of water in specific areas of water supply networks. Minimum flows are measured at the most relevant time, usually at night, between 1.00 and 4.00 am, when water consumption is the lowest. Then, proper functioning of network equipment could be evaluated. As water saving devices in the network, such as flow regulators, should be considered in WSS management (Berger et al. 2017). Further, leakage management should be considered in the context of the current and forecast supply and demand balance for each water supply or water resource zone in a river basin, alongside water policy objectives and other water efficiency measures to control consumption and measures to increase available supplies.

The overall performance of a WSS can be assessed by selecting suitable performance indicators (PIs) (van den Berg and Danilenko 2010). The general concept of evaluating the performance of a WSS is to compare its performance with established benchmarks through cross-comparison with similar utilities (Alegre et al. 2016). Performance indicators enable managers to have a clear picture about water utility performance with regard to leakage reduction, including impacts on environment, resource efficiency, and cost-efficiency. The IWA Manual of Best Practice Performance Indicators for WSS in the list of reliable indicators that are available for real losses points first of all Infrastructure Leakage Index (ILI). ILI accommodates the fact that real losses will always exists, even in the very best and well managed distribution system. As the current pressure regime may not be optimal, ILI should always be interpreted with some measure of pressure and only used for tracking progress if all justifiable pressure management has already been completed. Under system current pressure management regimes, calculation “how low could you go” in Mm^3^/year by entering system infrastructure characteristics and pressure in the equation for Unavoidable Annual Real Losses (UARL) allow for technical comparison of leakage levels (Cavazzini et al. 2020). The unavoidable level of losses is determined, and the enterprise is trying to reach and maintain it with the corresponding technical condition (Ociepa 2016;). Leakage, also referred to as “Real Losses” or “Physical Losses,” is one of three components of Non-Revenue Water (NRW) in potable water transportation and distribution systems. The other two components of NRW—unbilled authorized consumption and apparent losses (theft of water and customer meter under-registration)—represent water which is taken but not directly paid for by customers. As indicated by other researchers, volume and PIs of NRW all vary in direct proportion to the system input volume (SIV), and this is critical for monitoring the level and PIs of NRW (Al-Washali et al. 2019). However, reducing water losses is not an easy process. This requires many necessary measures to minimize the water loss to about 8–10%, as required by IWA recommendations.

The purpose of the research is to adapt the methodologies for water losses evaluation applicable to small water supply system (SWS). Recently, many SWS have been suffering serious drinking water scarcity due to frequent droughts caused by climate change. Also, ground water resources shrink due to progressive contamination of groundwater sources with nitrates from agricultural sources. Many SWS have to invest in expensive treatment technologies, which makes the water produced very costly. Therefore, the only way to improve the situation is to monitor, analyze, and eliminate water losses. The research also puts forward the thesis that the implementation of IT systems such as SCADA could reduce the level of recorded water losses. The paper presents results of investigations performed in the SWS—Wołczyn—with the aim to require an appropriate level of information for the efficient operation, maintenance, and management of a WSS. The assessment of water losses has been done based on the analysis and evaluation of management practice for 6 years period.

## Materials and methods

### Methods for determining water loss

Water loss assessment was conducted using several performance indicators presented below. The first one is the percentage of the total loss computed directly from equation:

1 $$ \mathrm{UFW}=\mathrm{CARL}/\mathrm{SIV}\ast 100\ \left(\%\right) $$

where UFW is the percentage of the total loss (%), CARL is the Current Annual Real Losses (*V*_wl_) (m^3^/year), and SIV is the system input volume (*V*_in_) (m^3^/year).

The second indicator is individual real losses indicator, here expressed in terms of the formula:

2 $$ \mathrm{RLB}=\mathrm{CARL}/\left({L}_m+{L}_r\right)\ast 365\ \left({\mathrm{m}}^3/\mathrm{km}/\mathrm{d}\right) $$

where CARL (*V*_wl_) is the Current Annual Real Losses (m^3^/year), *L*_*m*_ is the length of the mains pipelines (km), and *L*_*r*_ is the length of connection pipelines (km).

The next one is the Infrastructure Leakage Index (ILI) that allows to compare and properly evaluate actions to reduce water loss and also tells us about the technical state of the network. ILI describes the quality of infrastructure management and is the ratio of Current Annual Real Losses to Unavoidable Annual Real Loss:

3 $$ \mathrm{ILI}=\mathrm{CARL}/\mathrm{UARL} $$

where CARL is the Current Annual Real Losses (m^3^/year) and UARL is the Unavoidable Annual Real Losses (m^3^/year).

The IWA workgroup suggested that indicator ILI should be around 1.0 for the systems with very low water losses and could go above 5.0 for high leaking systems. Unavoidable Annual Real Losses (URAL), those whose removal is economically unjustified, is calculated by summing up the three components:

- Leaks unavoidable on main lines and lines without connections, expressed as 18 dm^3^/km/day/m of pressure
- Leaks unavoidable at connections to edge of street, expressed as 0.8 dm^3^/connection/day/m of pressure
- Leaks unavoidable at edge of street to customer meter, expressed as 25 dm^3^/km/da/m of pressure

This indicator is determined by formula:

4 $$ \mathrm{UARL}\kern0.5em =\left(18\times \left({L}_m+{L}_r\right)+0.80\times \mathrm{Np}+25\times {L}_p\right)\times P\times 0.365\ \left({\mathrm{m}}^3/\mathrm{rok}\right) $$

where UARL is the Unavoidable Annual Real Losses (m^3^/year), *L*_*m*_ is the length of the mains pipelines (km), *L*_*r*_ is the length of connection pipelines (km), *L*_*p*_ is the total length in km of underground connection pipes (between the edge of the street and customer meters) (km), Np is the number of service connection, *P* is the average operating pressure in system (here 38 m H_2_O), and 0.365 is the conversion factor for year and m^3^.

Other PIs are percent of Non-Revenue Water (NRW) calculated as follows:

5 $$ \mathrm{NRW}=\mathrm{SIV}\hbox{--} \mathrm{BW}/\mathrm{SIV}\ast 100\ \left(\%\right) $$

where NRW is the Non-Revenue Water (%), SIV is the system input volume (*V*_in_) (m^3^/year), and BW is the Billed water (*V*_bw_) (m^3^/year).

Water losses and unbilled authorized consumption make the NRW high. The main task for every water utility is to reduce NRW. The calculations were completed with the analysis of the obtained indicators. The average values from the years 2013 to 2016, when the IT tools in SWS did not function, were compared to 2018—the first full year of it operation in SWS. The year 2017 was deliberately omitted in the analysis of indicators, because it was the year in which SCADA (SCADA PRO-2000 2003) system has been installed.

**Fig. 1:**
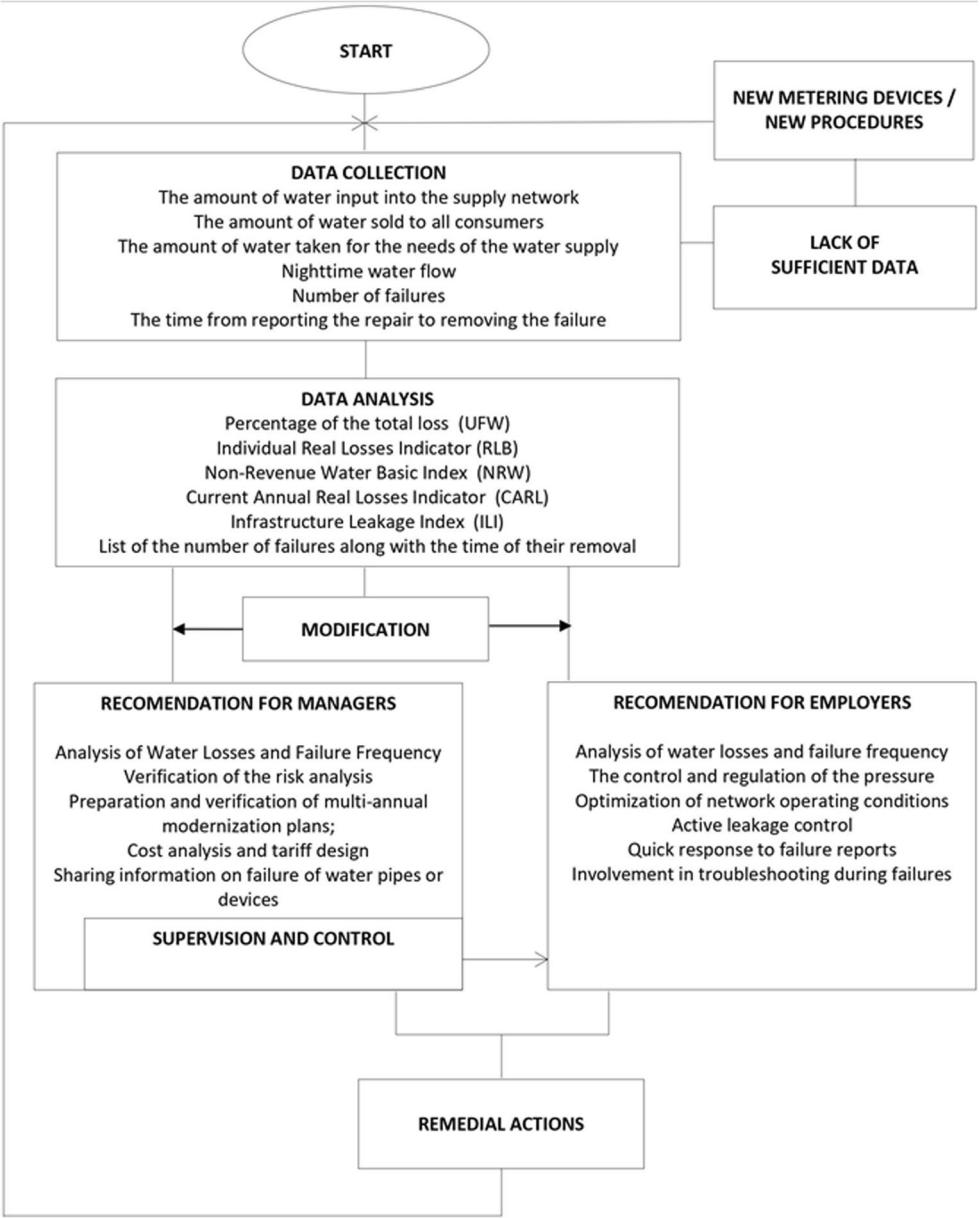
Workflow of the WSS analysis

The overview of the workflow along with the calculated PI is presented in Fig. 1.

The presented workflow combines indicator analysis with a management system based on quick response to a detected failure. The analysis determines UFW, RLB, NRWB, and ILI. At the same time, the critical parameters from them are determined, for a particular WSS. Comparison of the obtained indices with the critical values is an important element of the WSS condition assessment. Similarly as in the Deming cycle, the described tool provides for planning activities, executing activities in accordance with the plan, checking the degree of execution of objectives included in the plan, and on the basis of conducted analyses and lessons learned, the so-called improvement, i.e., ideas and solutions to be included in new plans.

### Case study description

The analyzed small water supply system (SWS) is located in Wołczyn, commune in the northern part of the Opolskie Voivodeship in Poland. SWS Wołczyn takes the water from 4 drilled wells, which are used to extract water from sandy Quaternary formations. Since 2004, a constant increase in nitrate concentration has been observed. The intake is located in a town about 2.5 km north-east of the town center. It uses water resources in the amount of *Q* = 150 m^3^/h at a depression of 165.6–167.0 m above sea level according to the decision of the Opole Voivode No. OS.II-7520-6/10/96 of 4 July 1995. Due to the high concentration of nitrates, the water is treated in the process of ion exchange, which significantly affects the price of water.

Due to the decreasing water consumption, the daily water production has also decreased over the last 20 years (Table 1). During this time, there has been a decrease in production by more than 30%. This is related to the growing awareness of the population and the price of water.

**Table 1:** Daily water production in SWS Wolczyn in the years 1996–2018

Year	1996	1998	2002	2007	2012	2015	2017	2018
m^3^/d	1258	1223	1172	1072	995	950	915	1023

Admittedly, water delivered into the network meets all parameters set for drinking water standards, but this is a result of advanced methods of treatment and permanent monitoring of the quality. These actions result in a high price of water production, and any uncontrolled leakages can generate losses for SWS. The waterworks in the Wołczyn commune are a reliable supply system in drinking water for about 14,000 inhabitants (Table 2).

**Table 2:** Number of inhabitants using SWS Wołczyn commune in the years 2013–2018

Year	2013	2014	2015	2016	2017	2018
No. inhabitants	14,000	14,000	14,000	13,900	13,900	13,800

Length of the active main water supply network of the Wołczyn agglomeration is 21 km in total, including the municipal area of the commune, of 5.1 km, and a rural area of 15.9 km. Distribution network has a total of 117 km. A significant part of a network is rural commune terrain; they constitute 103.8 km. The remaining sections are in the urban area. The summary of the length of the water supply network and water supply connections is presented in the Table 3.

**Table 3:** Characteristic lengths for water supply distribution system

Year	*L*_*m*_ (km)	*L*_*r*_ (km)	*L*_*m*_ + *L*_*r*_ (km)	*L*_*p*_ (km)	*N*_*p*_ (pcs.)
2013	21	117	138	152.9	2513
2014	21	117	138	153.2	2528
2015	21	117	138	153.4	2542
2016	21	117	138	153.5	2547
2017	21	117	138	153.7	2561
2018	21	117	138	154	2585

Based on the table, a gradual increase in the length of water supply connections in the years 2013–2018 can be seen. The number of new connections is also increasing. On the other hand, the length of the main and distribution networks does not change.

**Fig. 2:**
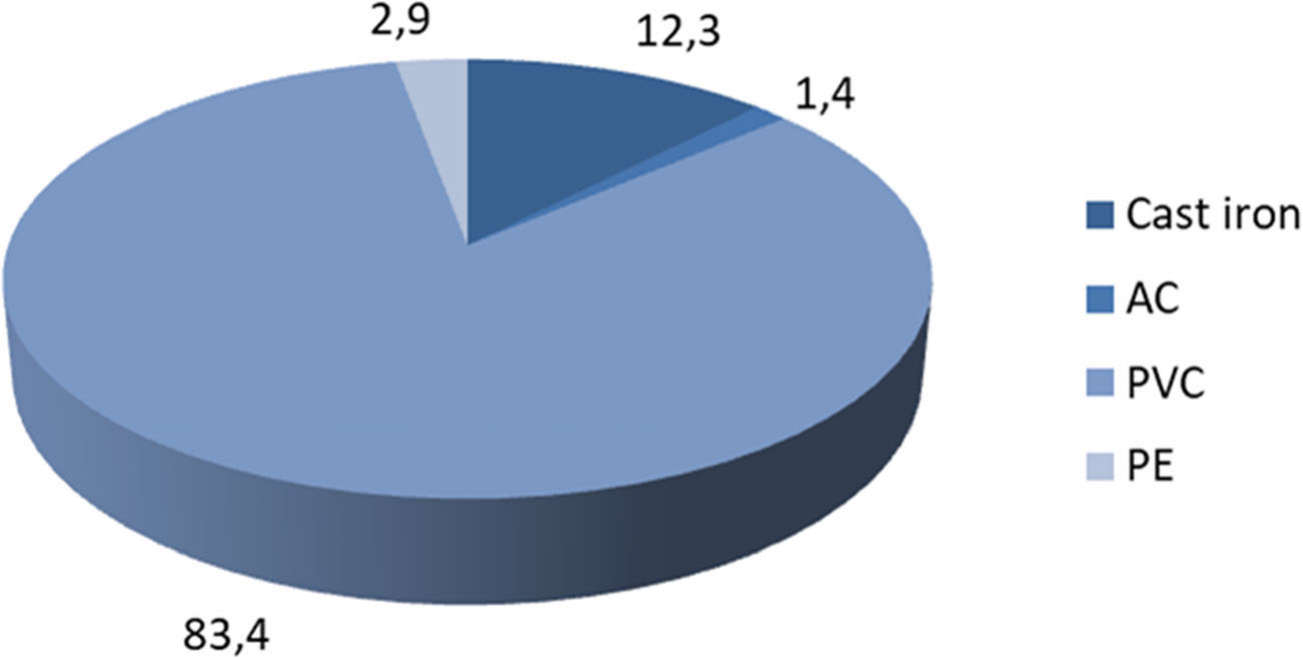
Material structure of the water supply network in the commune of Wołczyn

In the assessment of the failure rate of the water supply network and the amount of water losses, a very important parameter is the type of materials and the age of the pipes. The older the pipes are, the more likely they are to be damaged and therefore more likely to leak and cause water loss. The material from which they are made also plays a significant role. Some types are highly corrosive compared to others. Steel and gray cast iron pipes are characterized by the greatest damage intensity. They are the most vulnerable to corrosion and cracks. The diagram (Fig. 2) shows the types of water supply network materials in the Wołczyn commune.

Based on the graph (Fig. 2), it can be seen that the largest share in the material structure of the network includes pipes made of PVC. They are as much as 83% of all materials. Other pipes are made of cast iron, polyethylene, and asbestos-cement. This material structure results from the fact that the SWS Wolczyn was significantly modernized in the late nineteen decades.

Table 4 shows the material structure of the WSS in the Wołczyn commune depending on the period of operation.

**Table 4:** Water supply network by material structure and service life in kilometers

Water supply network	Up to 10 years	From 11 to 25 years	From 11 to 25 years	From 11 to 25 years	Total by material
			(km)		
Cast iron	-	-	16	1	17
Steel	-	-	-	-	-
AC	-	-	2	-	2
PVC	15	40	60	-	115
PE	4	-	-	-	4

Based on the data contained in Table 4, it can be concluded that the material structure of the pipes is systematically changing. A large part of the water supply network is between 11 and 25 years old. At that time, polyvinyl polyvinylchloride (PVC) prevailed. In recent years, pipes made of mainly polyvinyl chloride (PVC) have also been used. Only 4 km was made of polyethylene (PE).

The volume of water production and its sale depends on many factors, as well as the variability over time, years, months, weeks, or days. In Table 5, monthly water production, billed water, and water losses in Wołczyn SWS are presented for 2017 and 2018.

**Table 5:** Summary of the monthly water production balance in the SWS Wołczyn commune in 2017 and 2018

Miesiąc	System input volume *V*_in_	Billed water *V*_bw_	Billed water for domestic use *V*_*d*_	Unbilled authorized consumption *V*_unb_	Water losses *V*_wl_
	(m^3^/month)	(m^3^/month)	(m^3^/month)	(m^3^/month)	(m^3^/month)
2017					
January	40,500	33,498	28,682	4281	2721
February	36,273	32,075	27,219	2145	2053
March	41,372	32,891	26,294	4673	3808
April	40,770	33,621	27,100	3636	3513
May	43,050	35,590	28,361	4211	3249
June	45,431	38,132	29,418	3917	3382
July	43,621	36,334	29,524	4146	3141
August	44,227	38,421	30,654	2895	2911
September	43,158	36,897	28,948	3245	3016
October	40,989	35,573	28,179	2604	2812
November	37,136	34,076	28,434	1530	1530
December	39,276	34,045	27,901	2838	2393
2018					
January	38,511	33,539	28,080	2627	2345
February	35,742	31,759	26,701	2116	1867
March	39,458	32,856	27,500	3752	2850
April	46,129	36,594	28,806	5347	4188
May	51,250	38,996	29,814	6730	5524
June	51,576	42,028	31,601	5064	4484
July	53,878	38,448	29,483	8321	7109
August	55,539	40,934	33,213	7679	6926
September	49,924	39,367	30,894	5570	4987
October	48,552	37,477	29,780	5840	5235
November	43,727	38,692	30,210	2823	2212
December	43,582	35,048	28,037	4688	3846

In 2017, when total water production was 495,803 m^3^/year and total billed water was 421,153 m^3^/year, the highest level of losses occurred in March, while the lowest in November. Total water production in 2018 was 557,868 m^3^/year, while total billed water was 445,738 m^3^/year. The highest level of losses occurred in July, while the lowest in February. The amount of water sold to households and the water used for its own purposes is kept at similar levels throughout all of the analyzed years.

It is important to note that at the end of 2017, the SCADA-type intelligent management system was launched in the SWS Wołczyn. The SCADA-type system gives the possibility of control technological and random events on an ongoing basis. It was supposed that real-time knowledge, of the system state, and the ability to remotely control flows at critical points can vastly improve the performance of SWS.

Further calculations are made on annual data calculated on a monthly basis. A summary of the annual water production in Wołczyn for 6 years period is presented in Table 6.

**Table 6:** Annual water balance during 6 years period (2013–2018)

Year	System input volume *V*_in_	Billed water *V*_bw_	Billed water for domestic use *V*_*d*_	Unbilled authorized consumption *V*_unb_	Water losses *V*_wl_
	(m^3^/year)	(m^3^/year)	(m^3^/year)	(m^3^/year)	(m^3^/year)
2013	512,529	423,697	348,527	46,859	41,973
2014	538,619	444,407	342,887	50,853	43,359
2015	559,695	442,885	346,089	63,872	52,938
2016	534,173	429,663	344,678	59,087	45,423
2017	495,803	421,153	340,714	40,121	34,529
2018	557,868	445,738	354,119	60,565	51,573

When analyzing Table 6, it can be seen that the amount of water pumped into the network increased over the course of 6 years. In 2013–2018, an average of 533,114.5 m^3^/year was produced, the lowest in 2017, and the highest in 2015. Water demand by the inhabitants of the Wołczyn commune was variable in individual years. In 2018, water consumption increased probably due to the small amount of precipitation and drought. The years 2015 and 2018 are assessed as extremely dry, which translates into an increased amount of water sold. However, this does not affect the uninterrupted and adequate water supply. The average amount of water sold in 2013–2018 was 434,590.5 m^3^. The highest sales took place in 2018 and were higher by almost 2.6% than the average value for the analyzed period. The lowest value was in 2017 and was over 3% lower than the average amount. Water losses were not stable in the given years. They were on the average level of 44,965.8 m^3^. The smallest was 34,529 m^3^ in 2017, while the highest was 52,938 m^3^ in 2015.

## Results and discussion

The annual water loss is an important indicator in determining the management efficiency of WSS in a given area. A summary of all calculated water performance indicators for 6 years period on the basis of data obtained from the waterworks in Wołczyn is presented in Table 7.

**Table 7:** Calculated PIs for WSS

Year	UFW (%)	RLB_1_ (m^3^/km/d)	URAL (m^3^/year)	ILI (-)	NRW (%)
2013	8.2	0.83	63,951.07	0.66	17.33
2014	8.1	0.86	64,207.3	0.68	17.49
2015	9.5	1.05	64,420.16	0.82	20.87
2016	8.5	0.90	64,505.57	0.70	19.56
2017	7.0	0.69	64,718.44	0.53	15.06
2018	9.2	1.02	65,069.28	0.79	20.10

Using formula (1), the UFW in individual years was calculated. The analysis of the water loss indicators for the Wołczyn commune shows that that the percentage losses calculated on the basis of the loss shares in relation to the volume of water injected into the water supply network in 2013–2018 ranged from 7.0% to 9.5%. Their average value was 8.4%. Based on the results (Table 7), the share of water losses in relation to the volume of water delivered into the network was the smallest in 2017 and the largest in 2015. The technical literature states that water losses should not exceed 15% as in Nord America or 12 % in Western Europe (Chini and Stillwell 2018).

**Fig. 3:**
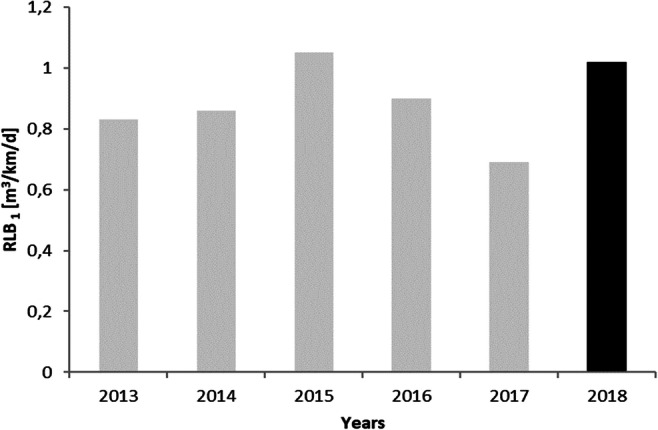
Real losses indicator RLB for 6 years period in Wołczyn (gray color, before SCADA implementation; black, after implementation)

**Fig. 4:**
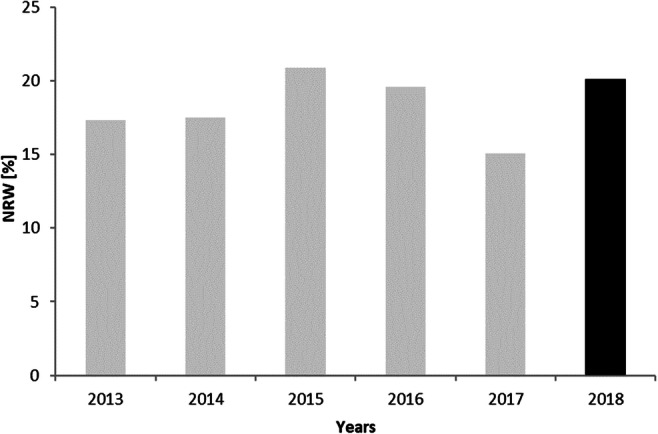
Non-Revenue Water indicator in Wołczyn system during 6 years period (gray color, before SCADA implementation; black, after implementation)

**Fig. 5:**
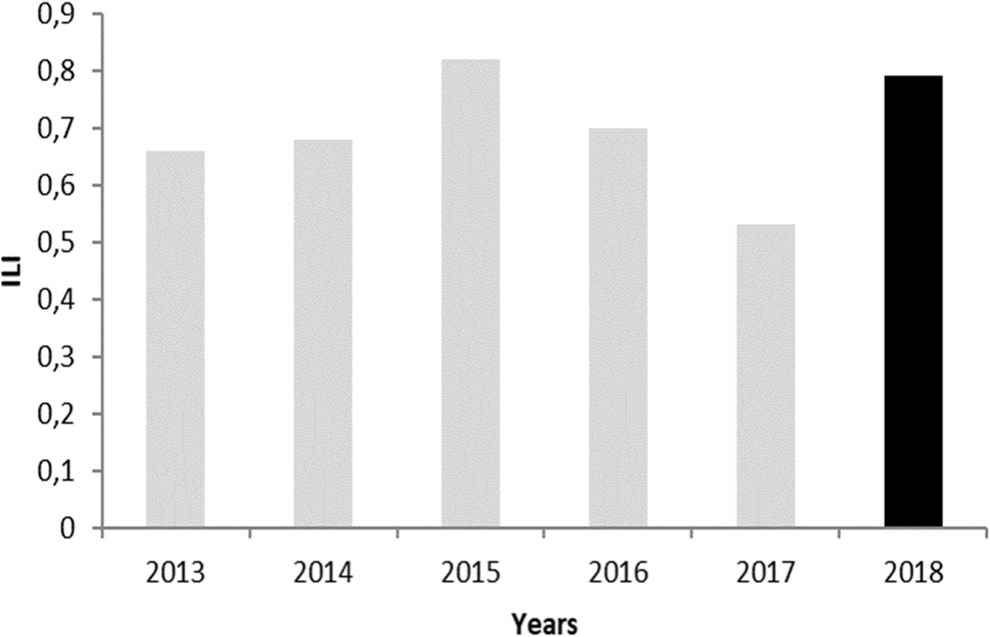
The Infrastructure Leakage Index (ILI, dimensionless) in Wołczyn system (gray color, before SCADA implementation; black, after implementation)

The URAL and ILI show a slightly increasing trend, although their values are not stable during the analysis period. It can be seen on the Figs. 3, 4, and 5. The ratio of the RLB only in 2015 and 2018 exceeds 1. In the remaining years, it ranges from 0.69 to 0.90. This indicates a very good condition of the water supply. The value of the NRW index remains on average at the level of about 20%. It includes both the volume of water used for technological purposes and water losses. NRW is most commonly used in developing countries and SM-WSSs, as it is easy to calculate and can be useful as a financial indicator (Kanakoudis and Tsitsifli 2010).

Extremely useful in order to assess the loss of water in WSS, there is also an ILI index, which in the Wołczyn commune ranges from 0.53 to 0.79. Determining this performance indicator makes it possible to assess the current state of the water supply network in a more specific way than the percentage. According to the criteria of the ILI value, specified by International Water Association (IWA), the condition of the WSS in the Wołczyn commune is described as very good. Also according the American Water Association (AWWA) and the WBI Banding System is in a very good condition for ILI values lower or equal to 1.5. General description of real loss performance management categories according WBI Banding System for BAND A: Further loss reduction may be uneconomic unless there are shortages; careful analysis is needed to identify cost effective improvement. Recommended actions according to the WBI Banding System for the water supply network in Wołczyn are the consideration of pressure management, introduction of better, active leakage control practices, and increased efforts in maintaining the network.

The analysis of the data presented in Fig. 3 demonstrates that the values of the unit as well as percentage water loss depend on the hydraulic loads indicator. The average value of the RBL equal 0.89 m^3^ km^−1^ d^−1^, against the background of Polish systems, indicates that the losses are maintained at a good level. Currently in Western countries, permissible actual losses are assumed lower than 1 m^3^ km^−1^ d^−1^ (Bergel 2012).

The volume of NRW, which includes apparent losses such as the uncertainty of water meter registration, was also analyzed. Calculating the NRW index allows to avoid distortions related to over-inflating the amount of water used for own purposes by some of the plants. In the value of this indicator, on the basis of Fig. 4, a downward trend can be observed for last 4 years, despite the existing deviations in year 2018. Mean value of about 18 % shows a good technical state of the network against the national data where the weighted average for this system size is about 24% (Haider et al. 2014).

An ILI, presented in Fig. 5, shows that the state of the network gradually increased, due to decrease of ILI. The decreasing value of the ILI indicator can be observed from 2015 to 2017, which demonstrates good management and improved efficiency of the water supply network in Wołczyn. It allows to evaluate whether losses under specified conditions of exploitation and at a given cost of lost water are at an acceptable or excessive level. This indicator is currently the most effective parameter for assessing the efficiency of water distribution. The analysis of loss indicators for the distribution system of Wołczyn shows that the plant has taken effective measures to reduce the leakage. Similar study results reveal that an additional decrease can be economically achieved by implementing pressure management and control measures on the homeowner side (Haider et al. 2019).

**Fig. 6:**
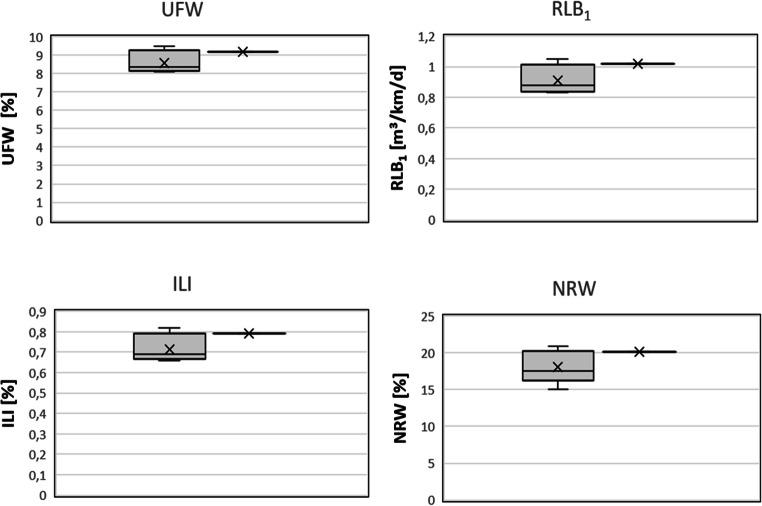
Graphical comparison of water loss indicators (gray, 2013–2016; black, 2018)

The last element of undertaken research was to check how the implementation of IT tools affected the level of losses in SWS. Graphical comparison of all calculated water loss indicators is presented on the Fig. 6.

As can be seen in Fig. 5, the implementation of IT tools has not improved the indicators. Each of the analyzed indicators in 2018 was higher than the average from 2013 to 2016. For the indicators, UFW, RLB_1_, NRW, and ILI, the values obtained in 2018 operate in the upper quartile of data for the years 2013–2016. URAL index from 2018 is significantly above the highest values obtained in 2013–2016.

## Conclusions

In view of the steady decline in water production and sales, the question of the level of losses becomes even more important; in general, water losses in SWS are currently one of the major operational problems of waterworks around the world. Water loss reduction is usually one of the most important tasks, which companies set themselves. Preventing leakage from the network is an inevitable element of management in a water company. Water loss analysis is the basis for taking management actions aimed at reducing the costs related to the distribution and production of water. Water losses are initially estimated using the balance method. In order to obtain a detailed assessment of them, the PI method is used, which allows obtaining information on the correctness and purposefulness of the actions taken. Based on the analysis, it can be noticed that in the years 2013–2018, water losses in the distribution system supplying the Wołczyn commune ranged from 7.0% to 9.5%. The non-profit water volume index averaged is around 18%. During the period considered, ILI was between 0.53 and 0.82. ILI is very useful in assessing the technical condition of the network. On its basis, the technical condition of analyzed SWS was determined as very good both according to the rigorous scope of the IWA and the American Water Association and the WBI Banding System, relating to highly developed countries.

The actual water losses are below the value of the non-profit water index, which proves that the distribution system is properly managed. Therefore, the presented analysis indicates a very good condition of the water supply network. The company is constantly taking steps to reduce the level of water loss. Among other things, by replacing water connections. Even though intelligent management system allowed, among other things, for the monitoring and pressure management, which made greater pressure stability possible, the detected level of water loss in SWS has not been decrease. SCADA does not recognize and thus does not signalize, automatically leaks. Therefore, the constant improvements of old water pipes and an expert control are necessary. There is no system that can detect small anomalies, so small leaks remain invisible for a long time. They can only be spotted when a serious failure occurs, which results in much higher instantaneous water flows.

## Data Availability

Not applicable.
